# Forecasting hourly foodservice sales during geopolitical and economical disruption using zero-inflated mixed effects models

**DOI:** 10.1080/02664763.2025.2519136

**Published:** 2025-07-07

**Authors:** Nathan A. Judd, Kalliopi Mylona, Haiming Liu, Andy Hogg, Tim Butler

**Affiliations:** a Department of Statistics, University of Warwick, Coventry, UK; b School of Mathematics, University of Birmingham, Birmingham, UK; c Department of Mathematics, King's College London, London, UK; d Electronics and Computer Science, University of Southampton, Southampton, UK; e Store Performance Limited, Hereford, UK

## Abstract

Accurate predictions of product sales are essential to the foodservice sector, for planning and saving of resources. In this paper, a zero-inflated negative binomial mixed-effects model with several factors was used to predict the total sales of different product categories, taking into consideration different sites, time and weather conditions. It fits quickly by maximising the ordinary Monte Carlo likelihood approximation. The model succeeded in accurate predictions with limited data where the random effects fitted well to the exogenous factors that added noise to the dataset. This enabled an improved inference from the model by reducing the variance in the estimates of fixed effects used in the interpretation of the results. This shows how statistical modelling, using less data, can improve predictions in the foodservice industry during times of volatile demand.

## 1. Introduction

The foodservice industry has been widely disrupted by COVID-19, which has impacted the supply chain, the labour availability and the customer demand. Customer demand will potentially continue to be volatile for years to come, driven by increasing geopolitical and economic factors such as the war in Ukraine, and labour and trade uncertainty.

Current research has focused on providing accurate forecasting using machine learning (ML) methods and artificial intelligence (AI) models, which rely on a large amount of homogeneous and/or heterogeneous data collected through a long period of time [6,9,11,17,25]. However, ML and AI methods in their current formulation perform poorly in periods of volatile customer demand. Therefore, alternative forecasting methods, using data from a shorter more homogeneous time-period, are needed. In [3], a detailed comparison between regression methods and pure prediction algorithms is provided, highlighting the longer-term validity of statistical modelling.

This paper aims to answer three research questions: Which features are useful for volatile forecasts? What forecasting methods are effective for use with short term and limited data, in the context of the food service? What variables are helpful for the prediction of sales?

We investigate the modelling approaches required to build functional demand models of different product categories as the response variable within real-life datasets. The data sets were collected from foodservice kiosks in city centre transit locations, over observation periods between four and twelve weeks. This provides much smaller datasets than the datasets previously used in the literature [9,17]. They cover a number of product categories sales, used as response variables in the models, such as hot drinks, cold drinks and sandwiches in a takeaway only environment. A number of independent variables, including weather, were available over several granularities of time.

## 2. Related work

Foodservice sales data can often be noisy, heterogeneous and over-dispersed. Potential variability range from the local-level, e.g. footfall proxies (arriving and departing trains), the weather and location [12,16,20], to the national-level, e.g. the unemployment rate and mean disposable income [8]. Furthermore, where causality exists, it may not be easily identifiable for modelling purpose – for example, concerts and sporting events. Machine Learning (ML) methods often over-fit to such datasets due to high correlations and low data quantity [19,22,25,26]. Furthermore, ML methods are not as robust in unstable environments where frequent monitoring of forecasting accuracy and re-modelling is advised [7,10,23]. The distribution of foodservice sales throughout the day and within the week is critical for store management. Allocating staff resources effectively can maximise profit by maximising sales and minimising costs. For example, accurate within-day forecasting could allow for opening more tills during busy periods and restocking and cleaning during quiet periods [23,24]. Similarly, accurate forecasting during the week could maximise sales via effective shift-allocation and could minimise costs via reducing product waste. Hence, accurate prediction of within-day and within-week demand is informational gold in the food service sector.

In [18], the importance of forecasting in food and beverage sales in staff canteens and restaurants for successfully managing restaurants and, from an environmental point of view, maintaining a low level of pre-consumer food waste is being identified. In this work, two Bayesian generalised additive models (GAMs) were proposed for the prediction of future values of the daily sold quantities of a given menu item. This approach requires only POS data; while other variables such as, weather information, special promotions, information regarding special events and holidays are not considered. In [2], uni-variate models synthesise dynamic generalised linear models for binary and conditionally Poisson time series, with dynamic random effects for over-dispersion This Bayesian forecasting methodology is being applied to supermarket sales forecasting. A Bayesian methodology for consumer sales forecasting of many supermarkets is also being presented in [1]. In [21], it was shown that multiple regression gives the most accurate predictions for daily dinner counts at a university dining center. ARIMA, exponential smoothing models and other econometric models are also broadly used in the related literature such as in [8,13,19]. The effects of weather factors are being considered in [5,23]. The former one uses stepwise regression and the latter one uses a machine learning approach.

In addition to the above, the weather company [14] that is part of IBM reported that a one-day forecast is normally accurate within 2.5 degrees Celcius, and a five-day forecast can accurately predict the weather 90 percent of the time, while a seven-day forecast can accurately predict the weather about 80 percent of the time. A ten-day forecast can only accurately predict the weather half the time. Since weather is an important factor to predicate the sales of different items. For example, there is a correlation between the sales of hot drink and the cold weather. The lack of accuracy of the weather forecast leads to the inaccurate prediction of the sales [5,15,20]. Therefore it is important the prediction model could make accurate prediction based on the short term data, for example within 5 days.

In this paper, it will be shown that accurate hourly foodservice sales prediction is possible in a variety of highly-heterogeneous settings, including transit hubs. This approach will be able to incorporate commonalities and discrepancies by site and by product. A by-product, by-site approach is found to be effective due to the large variation between sites and complex covariance matrix. Relationships between responses (product sales) and independent variables are close to being unique to each site in a brand and thus responsive to local conditions e.g. weather and footfall.

## 3. Methodology

Our methodology commenced with detailed analysis of the product sales transactions to identify its basic characteristics, potential significant variables and opportunities to apply either linear or non-linear approaches. In particular, the need to build and calibrate usable short term forecasting models with minimal datasets was paramount.

By examining plots of product sales by hour and day of the week, we identified potential significant effects. From this we tried fitting random effects to soak up within-day and within-week variance. Random effects for hour of the day, day of the week and the interactions thereof were found to be effective in forecasting and a large amount of variation was accounted for in this addition. Our approach was to use four weeks of training data. We found that the improvement in predictions was marginal when using eight weeks of data. Furthermore, due to unpredictability in regulations during government responses to COVID19 we believe this four-week approach is not only appropriate but advantageous in order to avoid overfitting to previous conditions. Calibration of the model was then employed to select variables and maximise accuracy. Forecasts, hindcasts and the investigation of non-standard training-testing set combinations were considered so as to maximise generality and account for all trends and influences.

### 3.1. Data characterisation

This study has utilised two datasets of transaction data. The first dataset covers a four-week period and encompasses three product categories and includes data for weather conditions and transit arrivals. The second dataset covers a 12-week period for a single product category, but also includes additional independent (control) sites in the same locations. Weather conditions are also included in this dataset, but not transit arrivals. The response variables, transactions by product category, are available at an hourly-total resolution, whereas, as mentioned earlier, variables such as weather and transit arrivals are available at a minute-by-minute resolution.

In Tables 1 and 2, we provide some descriptive characteristics of the independent variables and response variables of the first dataset, respectively, In Table 3, we provide some descriptive characteristics of the second dataset. In the next subsections, several probability density functions (PDFs) are provided as well for a more detailed explanation on the features of the data.

**Table 1.** Descriptive characteristics of the variables in the four-week dataset.

Stores	Weather	Temperature	Temperature band	Day part
1	Cloud	Min. : 0	0–5	Morning
2	Rain	Median : 7	6–10	Lunch
3	Clear	Max. :13	11–15	Evening

**Table 2.** Descriptive characteristics of the responses in the four-week dataset.

Hourly total sales	Hourly hot drinks sales	Hourly cold drinks sales	Hourly sandwiches sales
Min. : 0.00	Min. : 0.00	Min. : 0.00	Min. : 0.00
Median : 55.00	Median : 11.00	Median : 14.00	Median : 23.00
Max. :431.00	Max. :146.00	Max. :126.00	Max. :260.00

**Table 3.** Descriptive characteristics of the variables in the 12-week dataset.

Stores	Weather	Temperature	Temperature band	Day part	Hourly hot drinks sales
1	Cloud	Min. :−1.13	0–5	Morning	Min. : 0.00
2	Rain	1st Qu.: 8.24	6–10	Afternoon	1st Qu.: 1.00
3	Dry	Median :12.11	10–15	Evening	Median : 7.00
4		Mean :11.94	15–20		Mean : 11.97
5		3rd Qu.:15.35	Above 20		3rd Qu.: 15.00
6		Max. :25.26			Max. :128.00

#### 3.1.1. Four-week data

**Figure 1.**
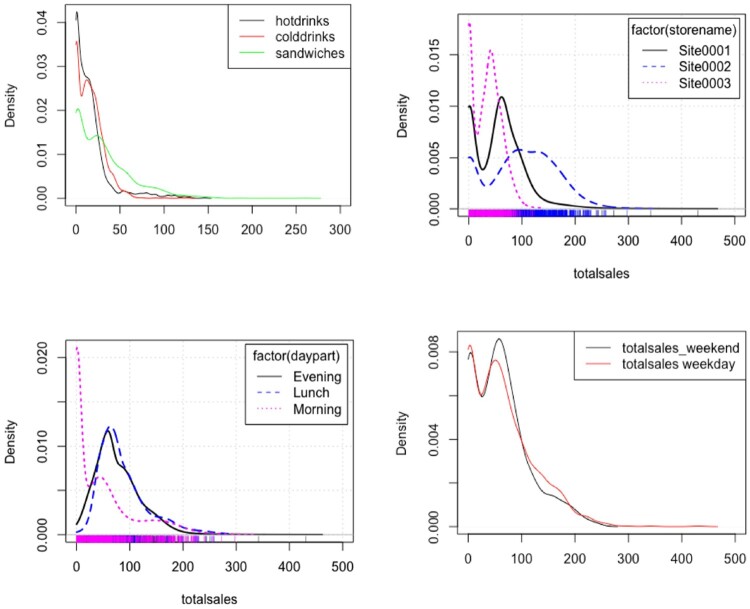
PDFs of total sales by different factors.

**Figure 2.**
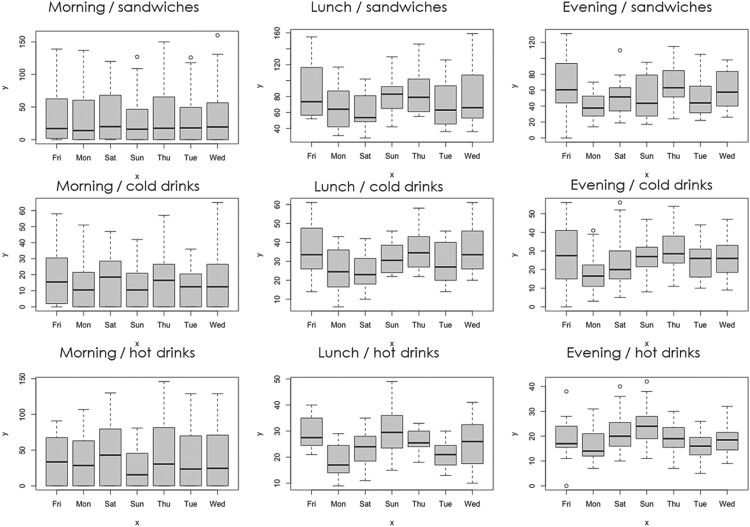
Box plots of the category sales for site 2 by day and day part for a four-week period.

From these data, we have summarised the observed transactions using probability density functions (PDFs) plots in Figure 1 and box plots for site 2 in Figures 2 (the box plots for other sites can be found in the supplementary material).

In particular the site summary's show significant temporal variability with influence from time of day, daypart, and day of the week (dow) for each product category studied. This clearly indicates potential significant variables for not only the site and product category, but also daypart and dow.

The PDF analysis indicates significant high-order content within the data alongside strong zero inflation. Modelling as a single population is clearly not warranted, and the overall characteristics will have to be matched by summing the contribution from each product category from each site by every daypart and day of the week.

Figure 1 shows the very high transaction rate for the sandwich product category and the very different characteristics for the second site (Site0002) compared to the other two sites. The morning day part also has a very different character from the lunch and evening ones, with the weekend being similar in character but not amplitude to weekday.

Figure 2 very clearly illustrates the influence of day of the week by product category and daypart for the second site (Site 2). This is different again for the first and third site, as illustrated in Figures 1 and 2 in the Supplementary Material.

#### 3.1.2. 12-week data

**Figure 3.**
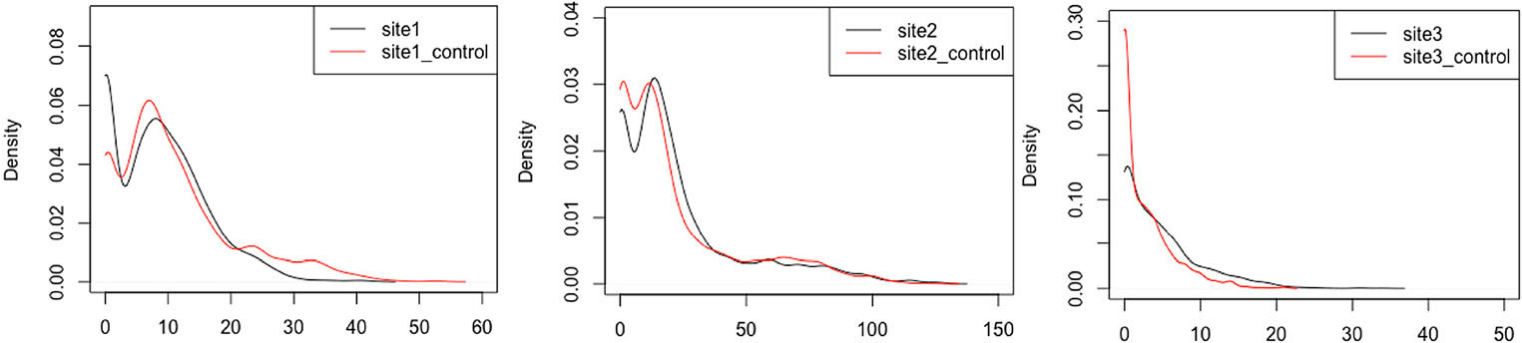
PDFs of total sales for hot drinks by different sites and their corresponding control sites.

**Figure 4.**
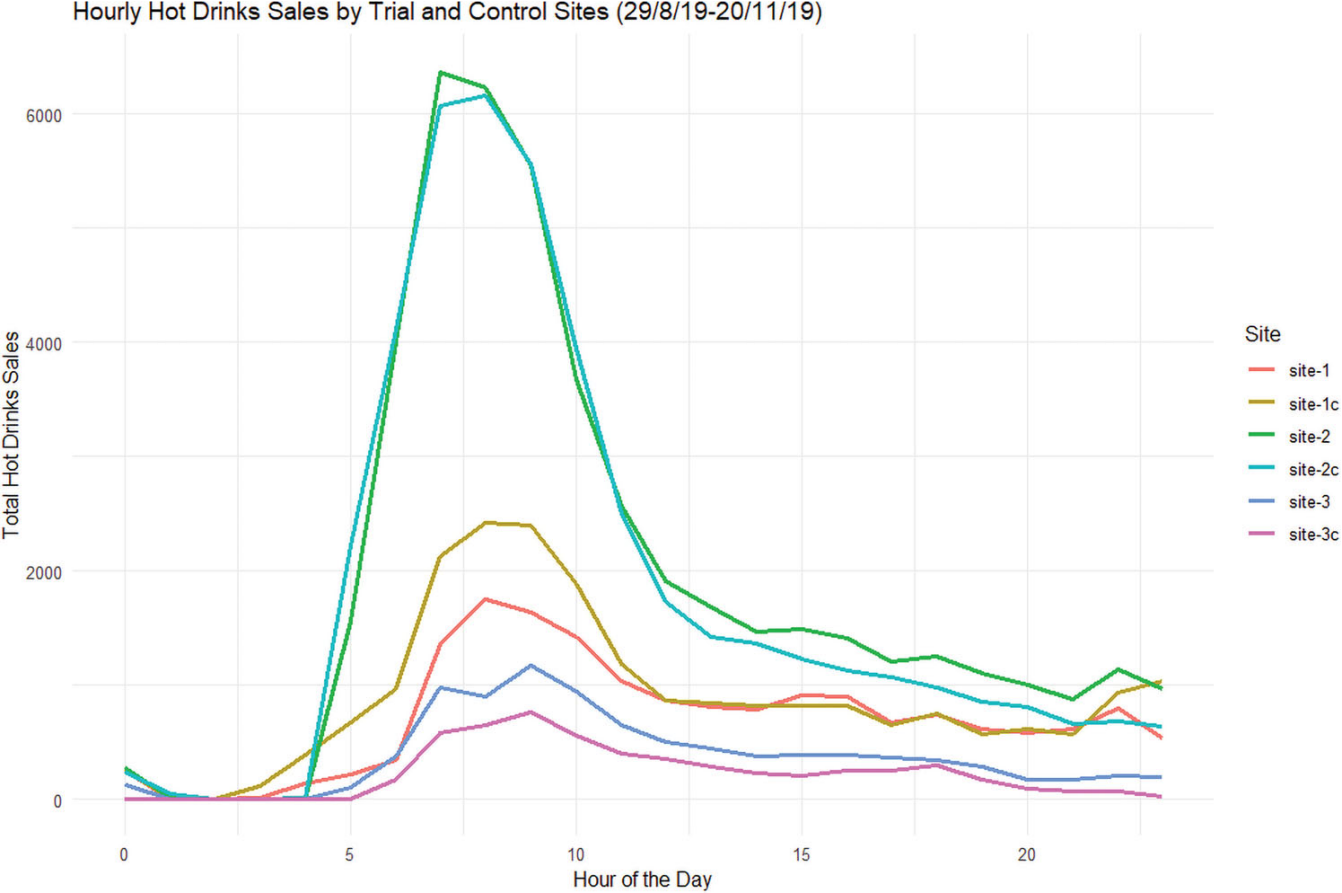
A plot of the hourly hot drink sales for the 6 sites (3 trial sites and 3 control sites) for the whole period (29/8/19 – 20/11/19).

**Figure 5.**
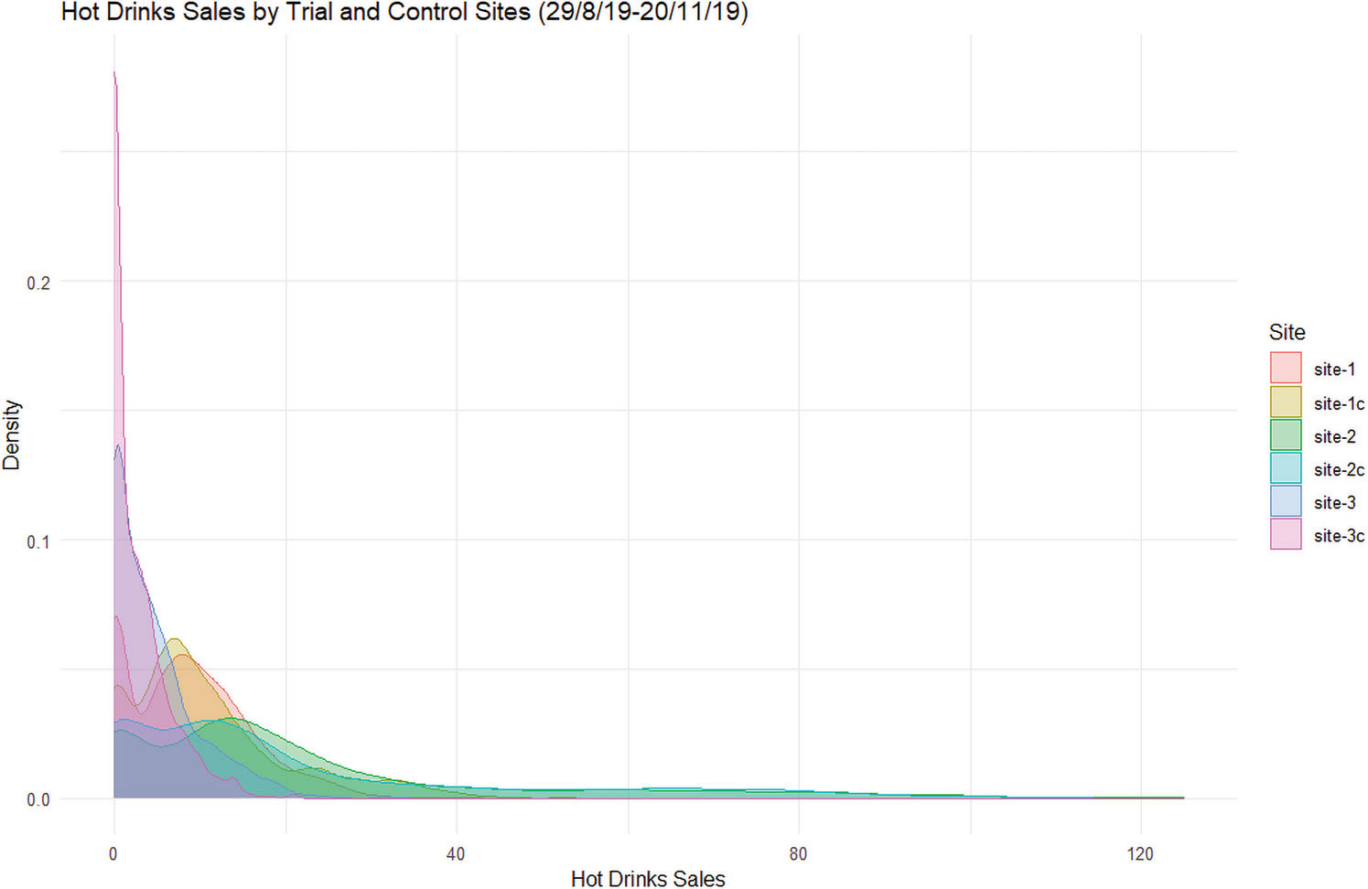
A plot of the probability density functions for the 6 sites (3 trial sites and 3 control sites) for the whole period (29/8/19 - 20/11/19).

**Figure 6.**
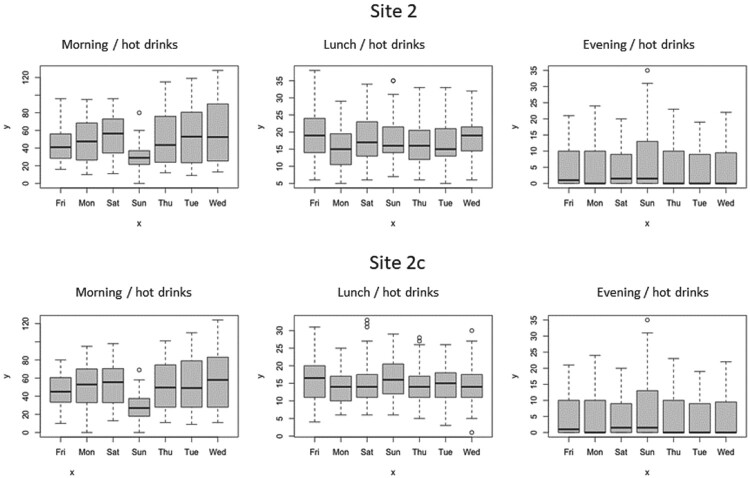
Box plots of the hot drinks sales for site 2 and its control by day and day part for a 12 weeks period.

These data are summarised as probabilities within Figures 3–5 (and Figures 3 and 4 in the supplementary material) and as box plots for the individual sites in Figure 6 (and Figures 5 and 6 in the supplementary material). The data patterns are clearly closest between sites and their controls, which are co-located but different brands, rather than across sites for the same brand.

Modelling the differences in sales characteristics between sites therefore becomes the major priority as the data clearly shows that the sales pattern is site specific. This suggests that position/branding within a site to be of lower significance than the footfall and hence sales character of the site itself.

The temporal characteristics for each site show a strong impact for both daypart (morning, lunch, evening) and day of the week for each site. The closest alignment between sites (and controls) is apparent in the lunch daypart with morning and evening showing less agreement.

### 3.2. Modelling approaches

In this section, through statistical modelling, estimates for several independent variables that might have an impact on sales are obtained. Both the Poisson and Negative Binomial distributions are useful for modelling since the dependent variables (hourly sales) are discrete. We run all the models in R, using the glmmTMB package [4].

#### 3.2.1. Models

Our main model is the negative binomial regression model. This model is similar to the regular multiple regression except that the response variable is an observed count that follows the negative binomial distribution. Thus, the possible values for the response are the nonnegative integers, which fits in our case where the response is the number of hourly sales. The negative binomial regression is a generalisation of Poisson regression which loosens the restrictive assumption that the variance is equal to the mean made by the Poisson model. The negative binomial model can take into consideration over-dispersion, which is when the dependent variable's variance is greater than the mean, whereas the Poisson model assumes that the variance is equal to the mean. A zero-inflation component is added to the models for both the Poisson and negative binomial dispersions to account for the excess counts of zero for the dependent variable. Some of the independent variables included in the model were the day of the week, the store, the temperature, the weather state and the day part.

#### 3.2.2. Random effects

Moreover, how can one account for additional sources of variability that are still unknown even if relevant confounding unit-level variables are controlled? Adding controls for random effects is one statistical strategy. Such models are called generalised linear mixed models (GLMMs). In that way, by dividing the heterogeneous units into homogeneous sets and controlling for variance in the dependent variable that occurs between those sets independently of the given independent factors, unnecessary variation can be eliminated. Importantly, the purpose of controlling for random effects is not to clarify their precise nature. Instead, the goal is to adjust for this random variance so that estimates of the specified independent variables are more accurate. The random effects that we have included in the model was the hour and the store (site).

#### 3.2.3. Alternative models and final choice

**Figure 7.**
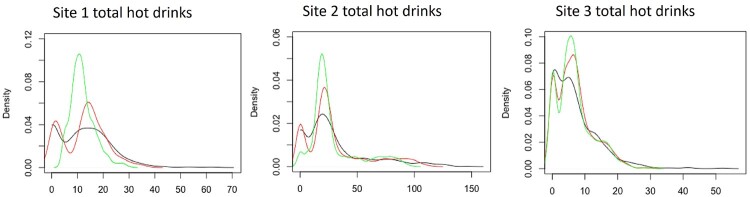
Plots of the PDFs for the four-week data for total hot drinks sales by site. The black line are the observed hourly total sales. The green line corresponds to the ZIP model with r.e. and the red line corresponds to the ZINB model with the same r.e.

Poisson models are often too restrictive in modelling count-data due to the mean equals variance assumption. It is common that this assumption is far too strong and thus the model may no fit well data which is over-dispersed. The negative binomial model (and its GLM counterpart) approximates the Poisson model well whilst introducing a second parameter linked to the variance. Other techniques to address overdispersion in the data include quasi-Poisson models, zero-inflated Poisson models and dispersion-adjusted Poisson models. Indeed, these models were tried and tested but found to be ineffective compared to negative binomial models. Zero-inflation was considered and was included in the final model but was not massively significant in dealing with overdispersion in general. The multi-modality and noisiness can also potentially be tackled using random effects in ‘mixed-effects’ GLMs. The issue of multi-modality natural leads one to consider mixing different Poisson distributions. This approach was considered but produced ‘bumpy’ probability density functions which seemed to skew predictions enough to make it an inferior approach compared to mixed-effects models. In Figure 7, we show the density functions for the four-week data for the total hot drinks sales by sites. In each subfigure, we can see a comparison between the observed density and the ones obtained using zero inflated Poisson (ZI-P) model with random effects (r.e.) and zero inflated Negative binomial (ZI-NB) model with the same random effects. Our model had included variables with respect to the weather, the sites, time and days of the week, while the random effects took into consideration the time and the site differences. From these graphs, we can clearly see the advantages of using a zero inflated Negative binomial model with random effects, especially for modelling the total hot drinks sales in sites 1 and 2.

In Table 4 we present the estimated coefficients with their standard errors for our alternative models for total sales. We also provide AIC and BIC values for model comparison. Similar tables for sales by product category can be found in the supplementary material (Tables 1–3). Table 4 shows that the zero-inflated Poisson GLMM is the best model of total sales. The random effects of both the zero-inflated negative binomial GLMM (ZI-NB GLMM) and the zero-inflated Poisson GLMM (ZI-P GLMM) improve the model fit as compared to the corresponding models without random effects (ZI-NB GLM and ZI-P GLM). Therefore, the coefficient estimates of the fixed effects are more accurate in the zero-inflated GLMM models than the zero-inflated GLM models. The pattern of significance is similar across the four models, with most day of the week being significant as compared to the Friday baseline. It is interesting to note that the GLMs produce more significant fixed effects that are not significant in the GLMMs. For example, temperature, day part and temperature band are all significant in the ZI-P GLM but not in either of the GLMMs. It may be that the randomness in hour and site correlate with these covariates and the fact that they are not significant in the GLMMs that fit better makes interpretation of the model and its dependencies clearer. It is also interesting to note that the same zero-inflated covariates are significant across all four models. This suggests that the randoness in hour and site do not correlate with the zero-inflation components of the day of the week. It also suggests that the day of the week is causing the extra incidence of zeros in total sales and that without zero-inflating a model, the coefficients of the day of the week coefficients (and other covariates) would be less accurate. The reasons for why day of the week is significant in both the incidence of zeros and strictly-positive integers representing sales, could be due to the changing commuting and footfall patterns during the time period.

**Table 4.** Model comparison (total).

	ZI-NB GLMM	ZI-NB GLM	ZI-P GLMM	ZI-P GLM
Fixed Effects				
Intercept	4.3321 (0.2919)***	4.2852 (0.0943)***	4.3110 (0.2767)***	4.3718 (0.0847)***
factor(month)November	−0.0161 (0.0356)	−0.0623 (0.0554)	0.0108 (0.0299)	−0.0677 (0.0444)
factor(dow)Mon	−0.3153 (0.0604)***	−0.2739 (0.0934)**	−0.2754 (0.0518)***	−0.2554 (0.0769)***
factor(dow)Sat	−0.0629 (0.0584)	−0.0586 (0.0903)	−0.1213 (0.0487)*	−0.0693 (0.0741)
factor(dow)Sun	−0.3538 (0.0624)***	−0.2967 (0.0965)**	−0.3187 (0.0548)***	−0.3733 (0.0821)***
factor(dow)Thu	0.1077 (0.0583).	0.1239 (0.0899)	0.0264 (0.0479)	0.0134 (0.0739)
factor(dow)Tue	−0.2321 (0.0588)***	−0.2324 (0.0910)*	−0.2058 (0.0492)**	−0.2021 (0.0733)**
factor(dow)Wed	−0.0815 (0.0592)	−0.0702 (0.0914)	−0.0723 (0.0489)	−0.0753 (0.0738)
temperature	0.0168 (0.0124)	0.0367 (0.0185)*	0.0100 (0.0107)	0.0498 (0.0161)**
factor(daypart)Lunch	0.1411 (0.1874)	0.1407 (0.0664)*	0.2085 (0.2069)	0.1473 (0.0532)**
factor(daypart)Morning	−0.1210 (0.1546)	0.0039 (0.0527)	−0.0642 (0.1713)	−0.2177 (0.0465)***
factor(temp_band)11-15	−0.1396 (0.1176)	−0.1280 (0.1770)	−0.0834 (0.1005)	−0.2599 (0.1537).
factor(temp_band)6-10	−0.0577 (0.0724)	−0.0687 (0.1089)	−0.0303 (0.0607)	−0.1667 (0.0944).
Zero-Inflated Effects				
Intercept	−2.3702 (0.3307)***	−2.3720 (0.3312)***	−2.3703 (0.3307)***	−2.3703 (0.3307)***
factor(dow)Mon	−0.7299 (0.5642)	−0.7355 (0.5672)	−0.7321 (0.5650)	−0.7325 (0.5652)
factor(dow)Sat	−2.3494 (1.0586)*	−2.3650 (1.0754)*	−2.3772 (1.0855)*	−2.3490 (1.0582)*
factor(dow)Sun	−1.1357 (0.6776).	−1.1353 (0.6786).	−1.2535 (0.7354).	−1.1336 (0.6766).
factor(dow)Thu	−17.7591 (2220.3923)	−17.5441 (1995.8761)	−17.8695 (2346.4375)	−17.7202 (2177.6386)
factor(dow)Tue	−17.6864 (2043.0913)	−17.6217 (1979.8205)	−17.7386 (2097.1287)	−17.9780 (2363.8761)
factor(dow)Wed	−2.3688 (1.0601)*	−2.3854 (1.0779)*	−2.5004 (1.1959)*	−2.3675 (1.0589)*
Random Effects				
hour (Variance)	0.08477	0.1056	0.1056	0.1056
site (Variance)	0.20753	0.1750	0.1750	0.1750
Model Fit				
AIC	7683.1	8321.5	7593.7	8318.5
BIC	7790.8	8419.8	7701.4	8416.9

## 4. Modelling results

Our chosen model was the zero-inflated negative binomial model with random effects. The reasons for this choice is its better quality in estimating the peaks and tails of the PDFs as demonstrated in Figure 7 and the small differences in AIC and BIC criteria with respect to the ZI-P GLMM. The final model included variables such as the day of the week, the store from which the data are provided, the daily temperature, weather characteristics of the day, such as rainy, cloudy or sunny weather, identification of the store as control or trial, and the part of the day.The random effects where the time of the day, the store and the part of the day, while the zero-inflation was based on the closing times of each store.

To note here, that the estimated model parameters were obtained using actual data e.g. for the day temperature. In practice, most probably estimated temperatures will be imputed in the model to make predictions. However, this will not be an issue, since the size of the estimated coefficient for the factor temperature is small, and this factor is not statistically significant. For example, for the four-week data the estimated coefficient of the temperature for the total sales is 0.0168. We can interpret this number as follows: for a one unit change in temperature, the difference in the logs of expected counts of the total sales is expected to increase by 0.0168, given the other predictor variables in the model are held constant. Hence the impact of using for examples two weeks in advance predicted temperatures and not the actual ones is small in the predictions of sales.

The ability of the final model to be used to make predictions is being evaluated at the following subsection.

### 4.1. Model calibration

**Figure 8.**
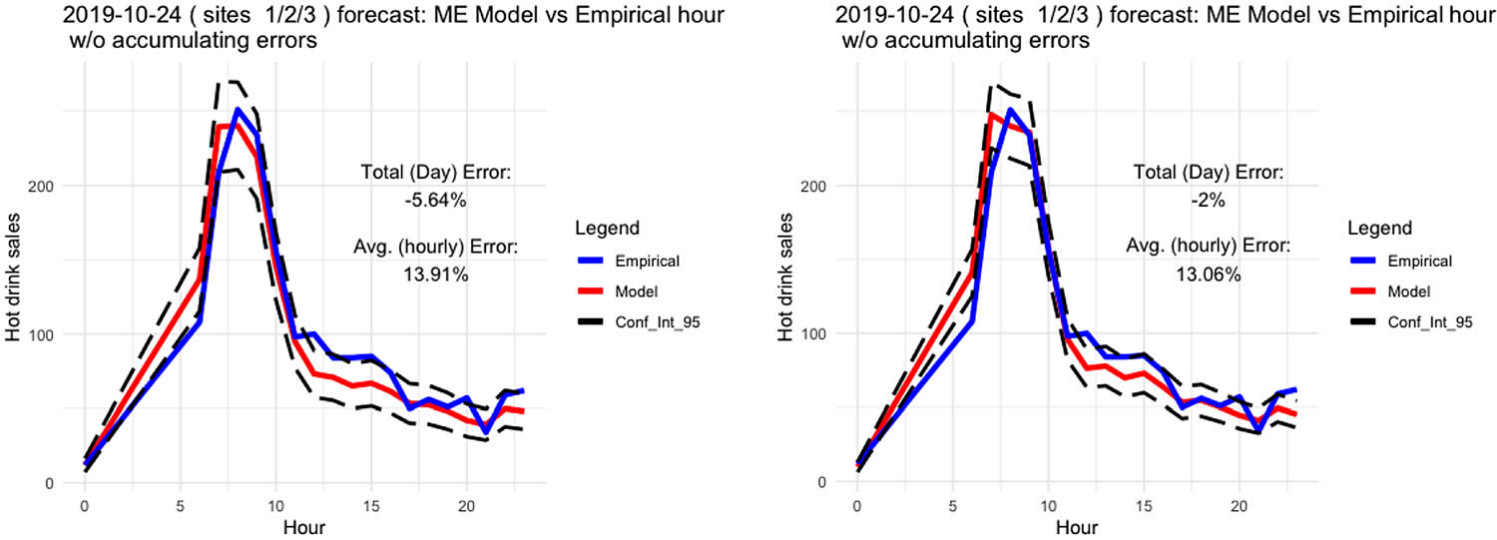
One day hourly prediction using four weeks data (left) and eight weeks data (right).

Since the dataset for the total sales of hot drinks included 12 weeks, we used it to evaluate the predictive performance of our model. Specifically, we used the first four weeks and the first eight weeks of data to predict a specific day included at the 12th week of data to check how well the model can perform with a few data. In Figure 8, we can clearly see how good the predictive performance of the proposed model is even with four weeks of data. This clearly demonstrates the advantage of the proposed method, against the data intensive requirement of several machine learning techniques.

**Figure 9.**
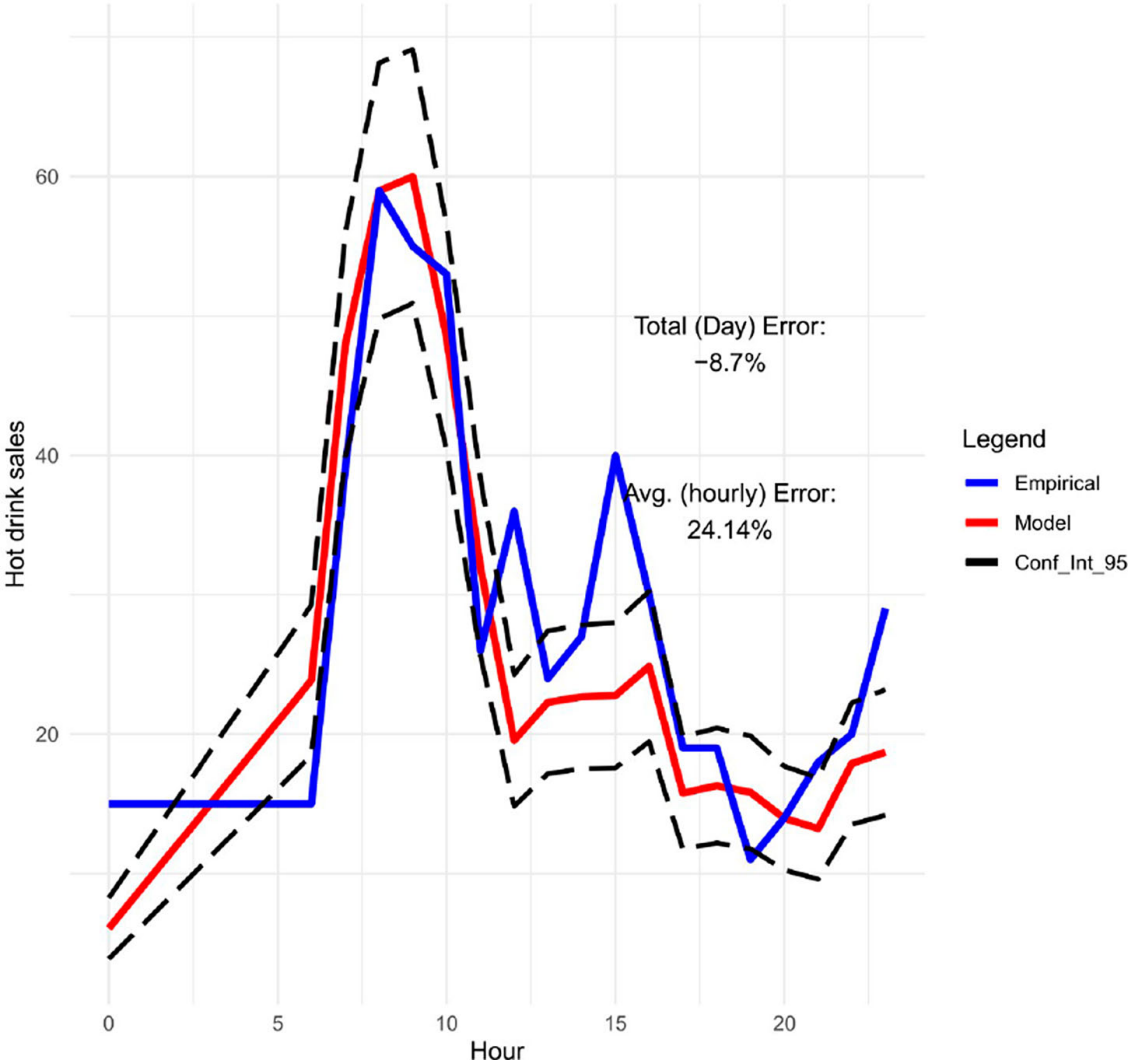
Hourly predicted vs actual sales for hot drinks with 95% confidence intervals (site 1 on 25/10/19).

**Figure 10.**
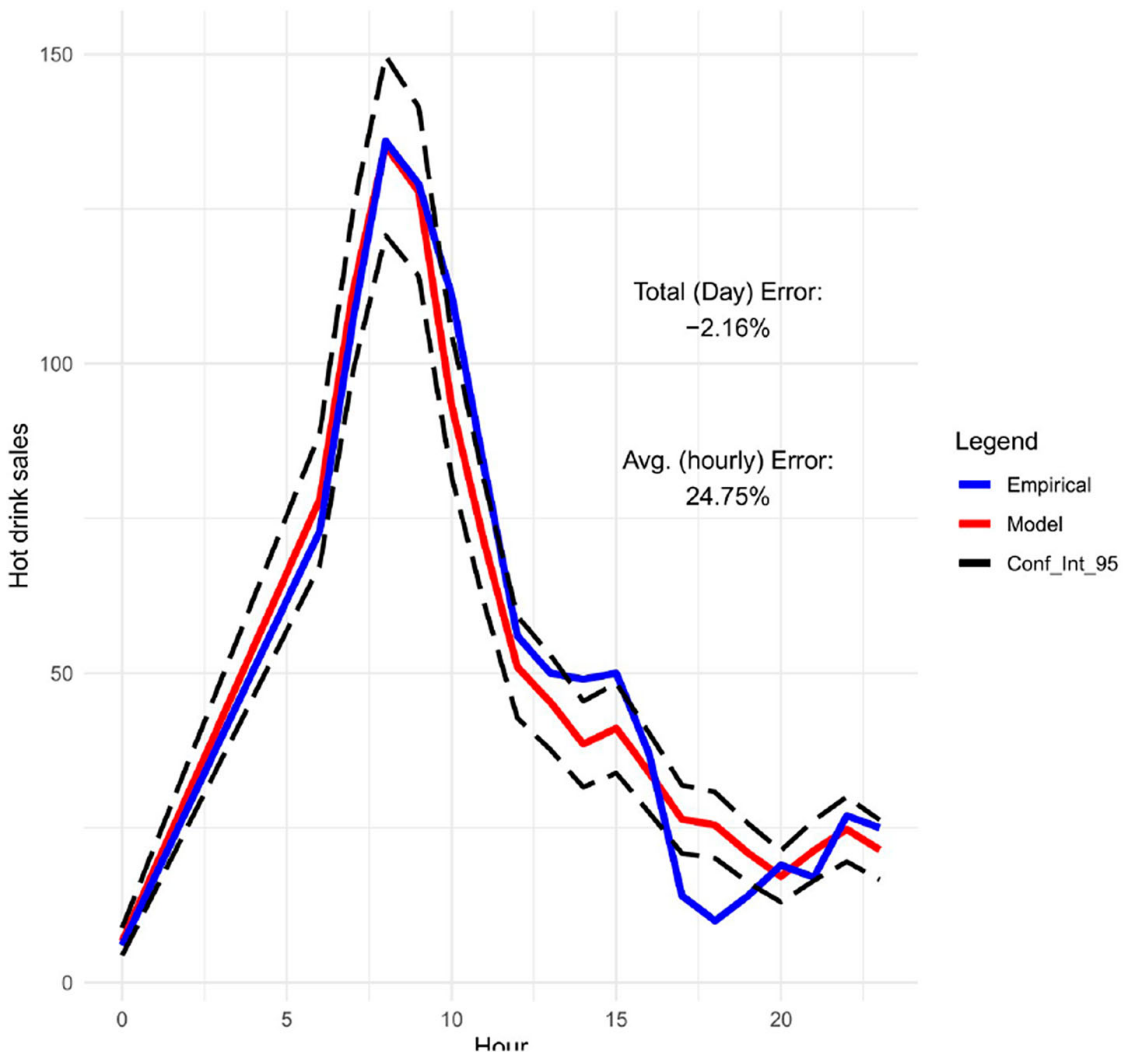
Hourly predicted vs actual sales for hot drinks with 95% confidence intervals (site 2 on 25/10/19).

**Figure 11.**
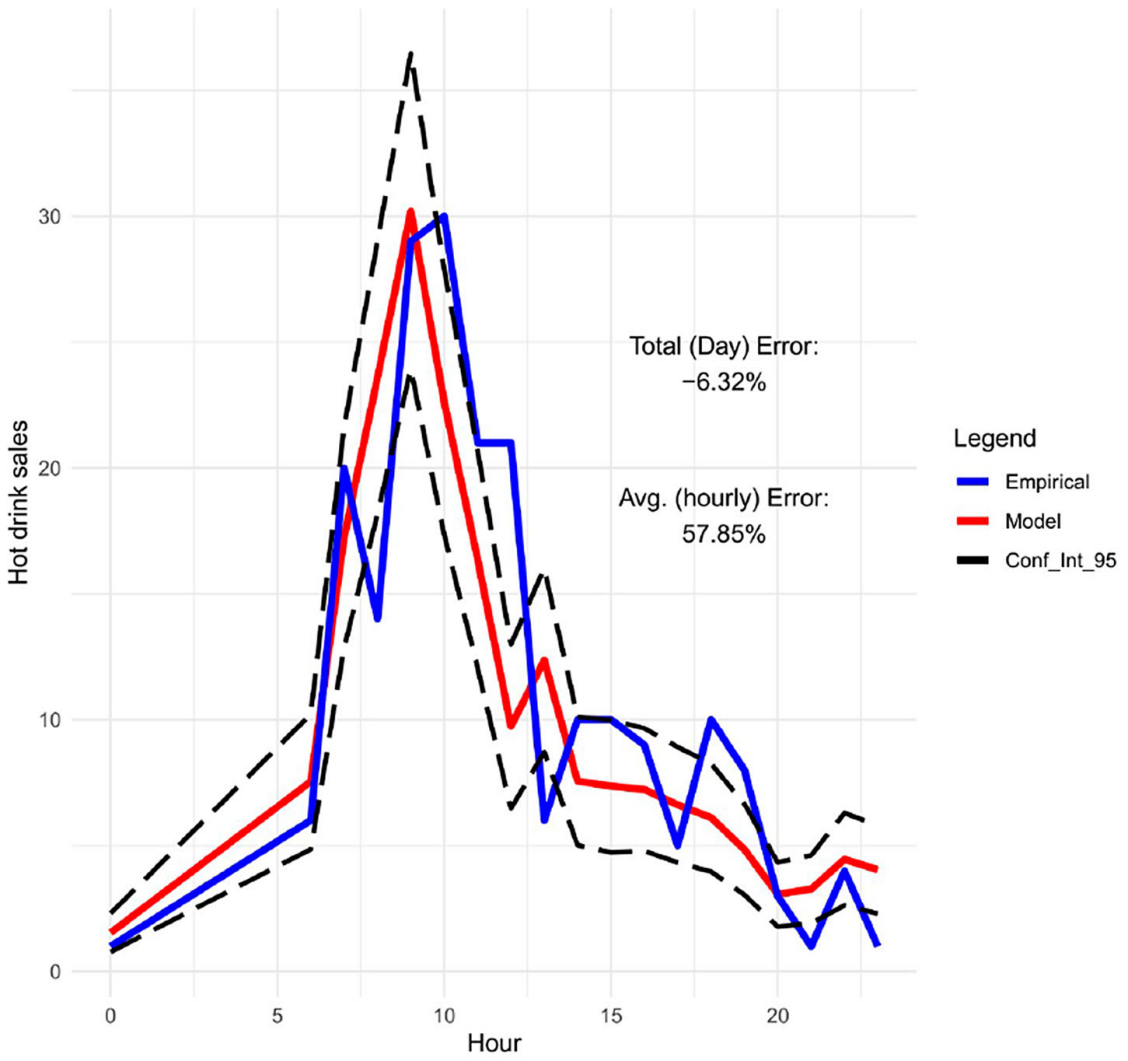
Hourly predicted vs actual sales for hot drinks with 95% confidence confidence intervals (site 3 on 25/10/19).

**Figure 12.**
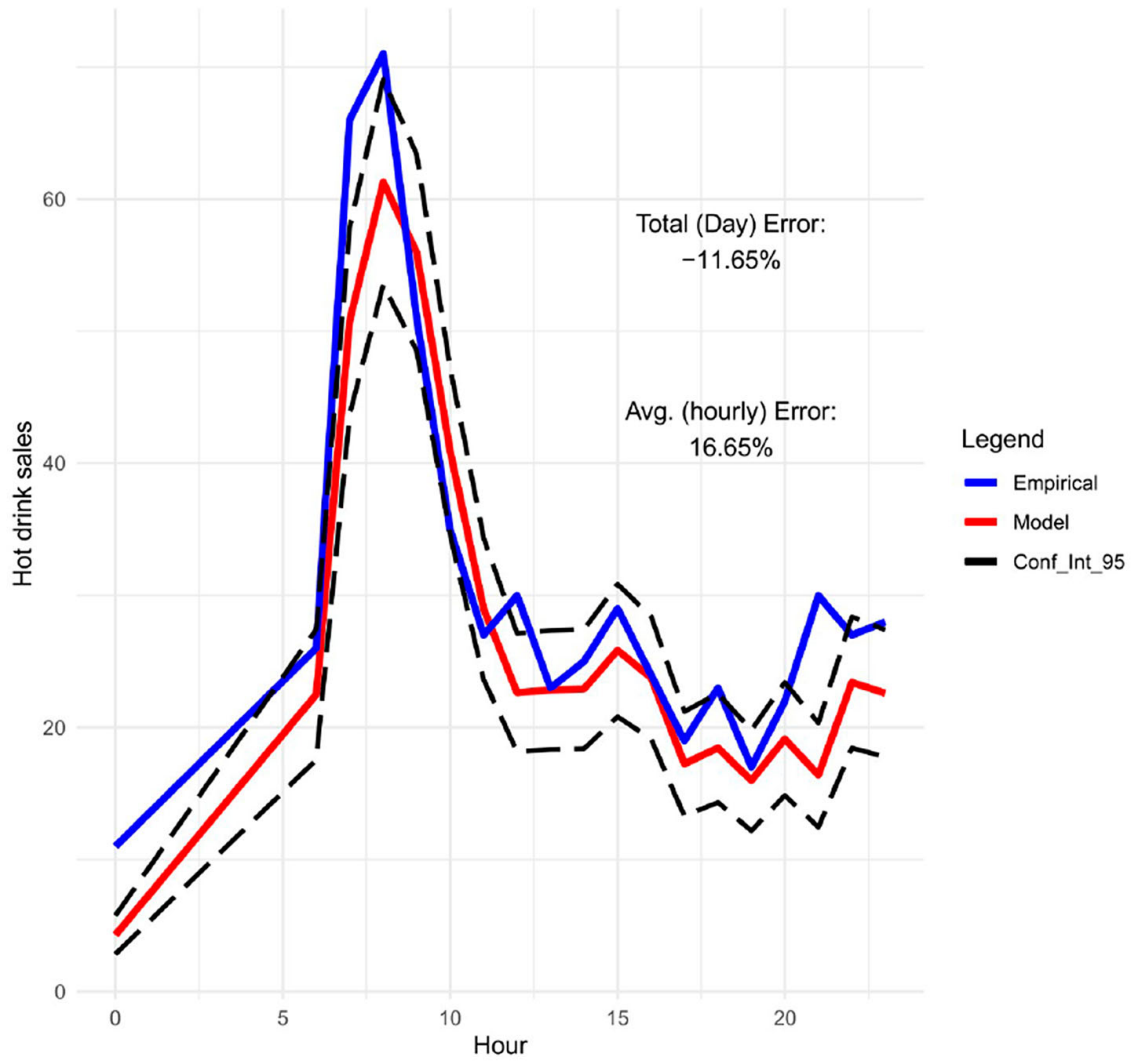
Hourly predicted vs actual sales for hot drinks with 95% confidence intervals (site 1 on 7/11/19).

**Figure 13.**
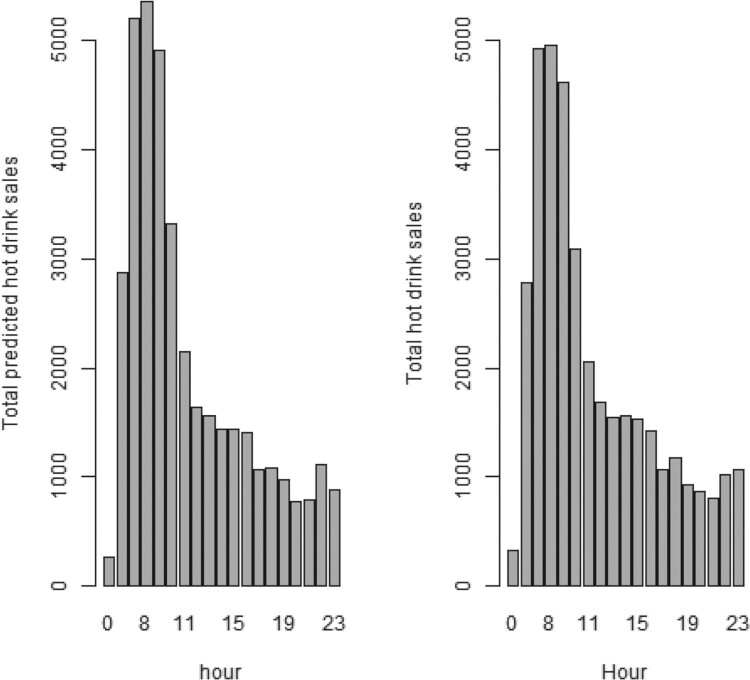
Hourly predicted and actual hot drink sales from Monday to Friday

**Figure 14.**
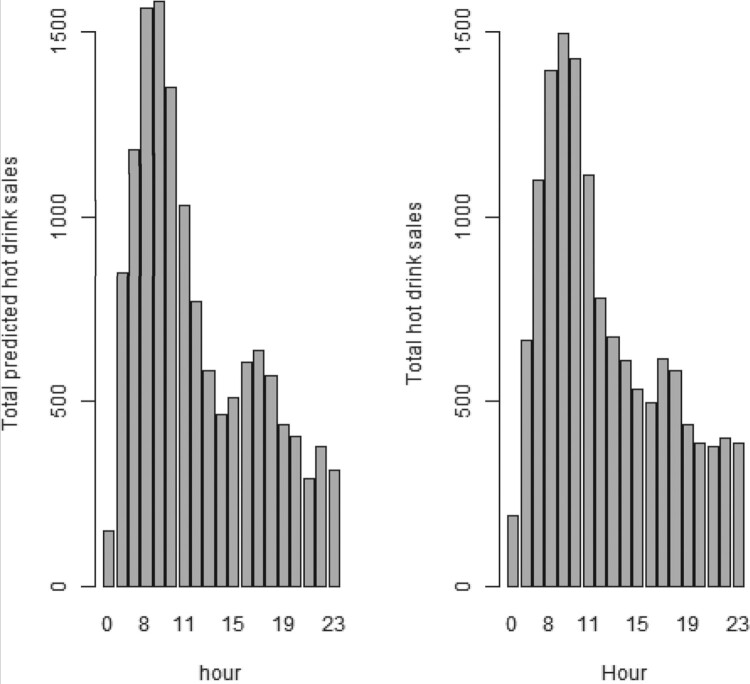
Hourly predicted and actual hot drink sales during the weekend.

Moreover, in Figures 9–12, we forecast the hourly hot drink sales of a specific day in different sites. When we compare the predictions to the actual hourly sales, we can clearly see how well the model performs in predicting the maximum sales and the hourly sales overall. In order to check the prediction performance of the model during the working week and on the weekends, in Figures 13 and 14 we provide the hourly predicted and actual hot drink sales, separating by weekday-weekend.

## 5. Discussion

### 5.1. Weather

The Lorenz limit to conventional weather forecasting has been shown by previous MIT work to be about two weeks. This thereby establishes a practical limit to accurate sales forecasts as we have shown weather to be a key (but not the only necessary) variable in our modelling. Moreover, the confidence limits for modern weather forecasting are not well documented in the literature for comparison. Weather and daypart were found to be significant in predicting sales of hot drinks and cold drinks. They were not significant for total sales. The GLMM structure was able to disentangle temperature and daypart as proxies for footfall from their direct impact on individual product categories. This also shows that modelling product categories individually is superior to modelling all product categories using the same model.

### 5.2. Modelling utility

Our modelling results – given actual weather state – can provide useful forecasts around the 95% confidence limits for a combination of site, product category and day. Therefore we can show a level of utility for sales forecasting consummate with the limitations documented for weather forecasts.

### 5.3. Volume of data

Our modelling results have shown that utility can be taken from calibrating the model with only four weeks data but there are minor improvements available by using eight weeks if available (Figure 13). This was a key objective for this work, building a usable alternative pathway to Machine Learning's requirement for ‘bulk’ data [9].

### 5.4. Site by site

The modelling and data analysis has clearly shown the need for a site by site, product category by category approach to sales forecasting in these environments. Transit hubs by nature are footfall heavy sites and their characteristics have been shown to be clearly different. The comparison between modelling for trial and control sites within an environment shows that the models are predominantly 'environment' models and the difference for the predicted product category between trial and control sites is secondary to the difference between environments (Figure 6). The addition of the random effects for site produced better fitting models, thereby indicating that site is important and a predictor of sales. This is consistent within product categories, as can be seen in the corresponding tables from the supplementary material.

### 5.5. Peak flow

The modelling results are showing very good competence for matching peak product category sales amplitude and timing – for a given site and day combination. This is key to the utility of the models, matching overall daily sales is secondary to forecasting peaks and timing for utility in scheduling staff and stock availability in any given daypart.

A significant difference between the ZI-NB GLMs and the ZI-NB GLMMs is the fact that temperature and daypart are significant in the former but not the latter. This suggests that the random effects of the GLMMs are correlated with temperature and daypart. Since the GLMMs fit better, we can conclude that hour and site are better proxies for footfall (and consequentially, of total sales and peak flow) than temperature and daypart. This interpretation holds overall, i.e. for the total sales, but not for the individual product categories of hot drinks, cold drinks and baguettes. Each product category has its own relationship with daypart and weather (either directly or via the proxies of hour and site). For example, Table 4 in the supplementary material shows that temperature and daypart morning (against the evening baseline) were significant (at the 10% significance level) for cold drinks in the best fitting model, which was a ZI-P GLMM. As would be expected, increases in temperature were associated with increases in sales of cold drinks. Similarly, the morning daypart was associated with a lower rate of cold drink sales as compared to the evening daypart. We conclude that overall hour and site are better proxies for footfall than temperature and daypart, but for sales within individual product categories temperature and daypart influence sales somewhat independently of footfall. These insights lead to superior modelling and forecasting of peak flow for total sales and individual product categories. For example, even if footfall is high, if it is a winter morning the sales of cold drinks are likely to be lower than the sales of hot drinks. This suggests that the GLMM approach is able to differentiate between direct causation and correlation via proxy status, leading to superior insights, modelling and forecasting. The fact that the total sales is unaffected by weather suggests brand loyalty and customers buy the product they prefer for the weather from the store they are loyal to, rather than buying hot drinks from one store and cold drinks from another.

### 5.6. Industry value

The modelling presented here can be useful for forecasting labour, inventory, promotions and attribution. It is perhaps the latter two uses where the lack of alternative approaches is most pressing. Attribution for any marketing or promotional action local to a site requires a good (95% confidence) forecast of what would have happened without that action.

Most pertinent to industry is the fact that the introduction of random effects is seen to reduce the scope of the significant fixed effects to only day part. This is a signal to industry that during periods of instability and changing consumer pattern, random effects lead to better model fits and more representative fixed effect coefficients of the variables of interest for inference. For example, Table 4 shows that all days other than Thursday were significantly different from Friday. Given the work from home directives during the study period, it suggests that there was a lower volume of footfall and thus sales during Thursdays and Fridays, which are at the end of the week and are intuitive choices of days to work from home, if available. Furthermore, the day of the week was significant in all models for the zero-inflated coefficients. This means that GLMs and GLMMs without zero-inflation are not suitable for modelling stores in transit hubs. This is likely due to the extended opening hours of stores in transit hub locations. GLMMs were able to distinguish between direct causation and proxy status, as demonstrated by the fact that temperature and daypart were not significant in total sales but they were in the individual product categories. A GLM would not be able to distinguish between two correlation proxies for footfall and disentangle its proxy status with direct impact on sales. Any modelling in related industries should consider zero-inflated models.

## 6. Conclusion and future work

Modelling and forecasting the volatile customer demand caused by increasing geopolitical and economic factors such as COVID-19, the war in Ukraine and labour and trade uncertainty is a challenging task, due to the short periods of stable demand. In this paper, we have demonstrated the first step towards a robust modelling approach for foodservice predictions of sales. The zero-inflated mixed-effects model was utilised for different product categories. It has accommodated several factors such as different sites, day of the week, hours, weather and temperature conditions. By using this model, we managed to accurately predict the peak flow and the tail of the distribution of sales, clearly evidenced at the provided figures. The mixed effects approach yields more accurate causal mapping between independent and dependent/response variables. Moreover, the modelling approach has been shown to work with only four weeks of data for calibration, significantly improving the utility of prediction over more common ‘big’ data approaches, such as machine learning algorithms often needing years of training data. Such accurate predictions with a small amount of data are essential for store optimisation during volatile customer demand. In addition, this model is able to account for changing commuter patterns such as from changes in the prevalence of working-from-home.

The scope of this work is clearly limited by the data available and sites/environments modelled. Further work is needed to expand the nature of the environments modelling – to high street takeaways for example – and additional product categories. This work is underway. The datasets used in this paper cannot be shared, for reasons of confidentiality. Nowadays, artificial intelligence (AI) offers tools to create similar datasets, however extensive research is needed on how to do this efficiently (e.g. number of data need to train the algorithm, amount of required human checks etc). This has also been included as part of our future work plans.

## Supplementary Material

Supplementary Material.pdf
