# Supplementary material for: Forecasting hourly foodservice sales during geopolitical and economical disruption using zero-inflated mixed effects models
Source: J Appl Stat. 2025 Jul 7;53(2):372–90. doi: 10.1080/02664763.2025.2519136 (PMC12872089; doi:10.1080/02664763.2025.2519136)
Supplement: Supplementary Material.pdf [file CJAS_A_2519136_SM4268.pdf]

# Supplementary Materials for
# Forecasting hourly foodservice sales during geopolitical and economical disruption using zero-inflated mixed effects models

Nathan A. Judd*[1,2], Kalliopi Mylona†[3], Haiming Liu‡[4], Andy Hogg§[5], and Tim Butler¶[5]

[1]Department of Statistics, University of Warwick, Coventry, CV4 7AL, UK
[2]School of Mathematics, University of Birmingham, Birmingham, B15 2TT, UK
[3]Department of Mathematics, King's College London, London, WC2R 2LS, UK
[4]University of Southampton, Southampton, SO17 1BJ, UK
[5]Store Performance Limited, Hereford, HR1 4SY, UK

## 1 Box plots for Sites 1 and 3 and PDfs for for product categories

*n.a.judd@bham.ac.uk
†kalliopi.mylona@kcl.ac.uk
‡h.liu@soton.ac.uk
§andy@store-performance.com
¶tim@store-performance.com

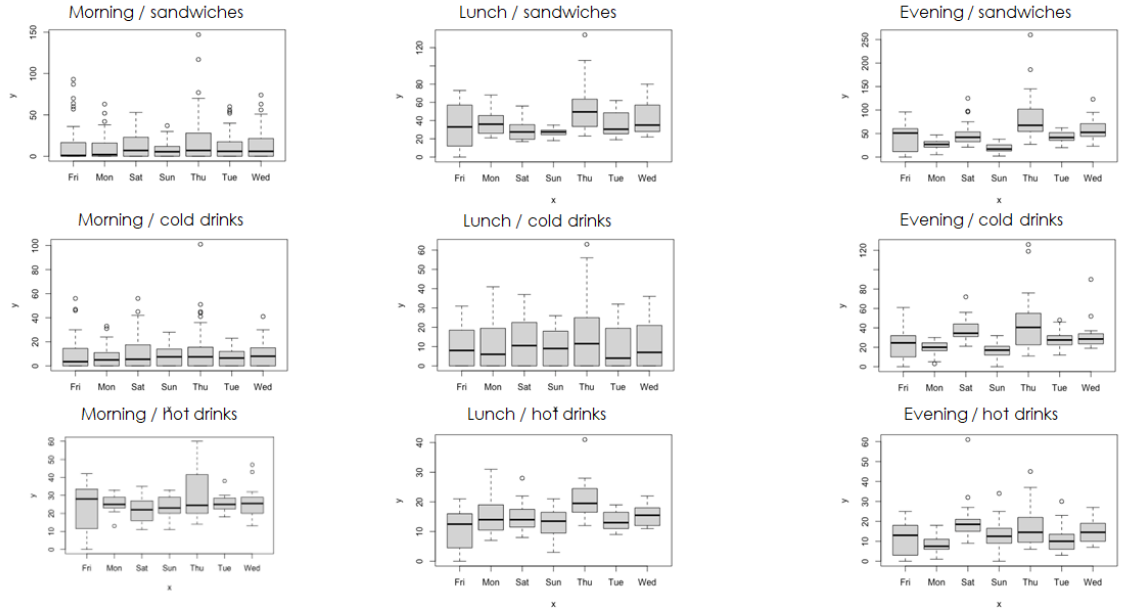


Figure 1: Box plots of the category sales for site 1 by day and day part for a four weeks period

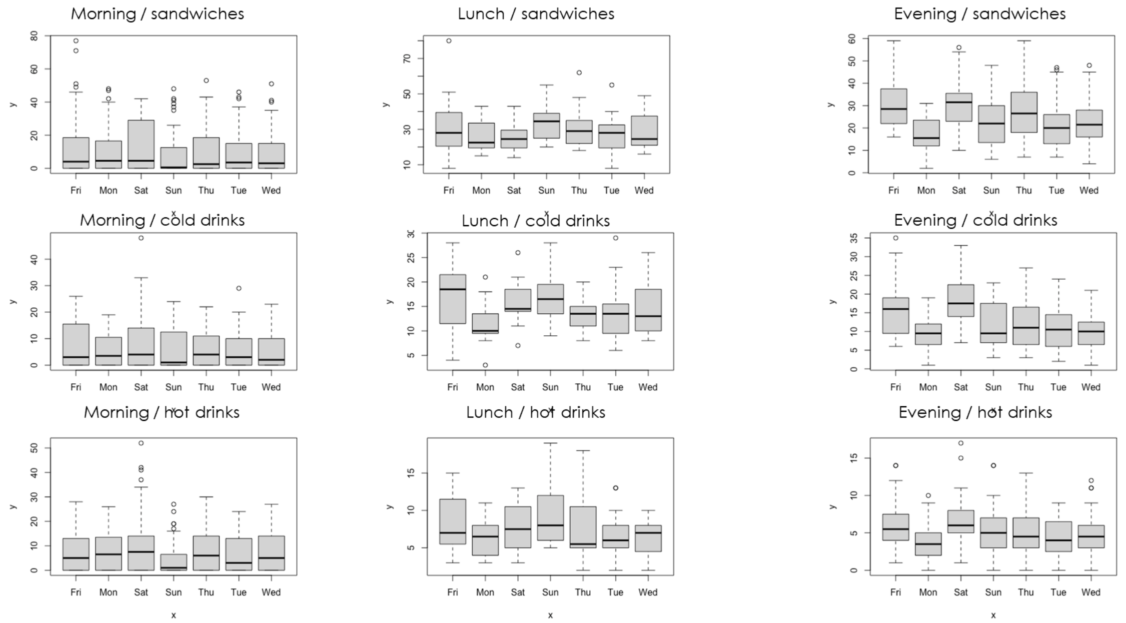


Figure 2: Box plots of the category sales for site 3 by day and day part for a four weeks period

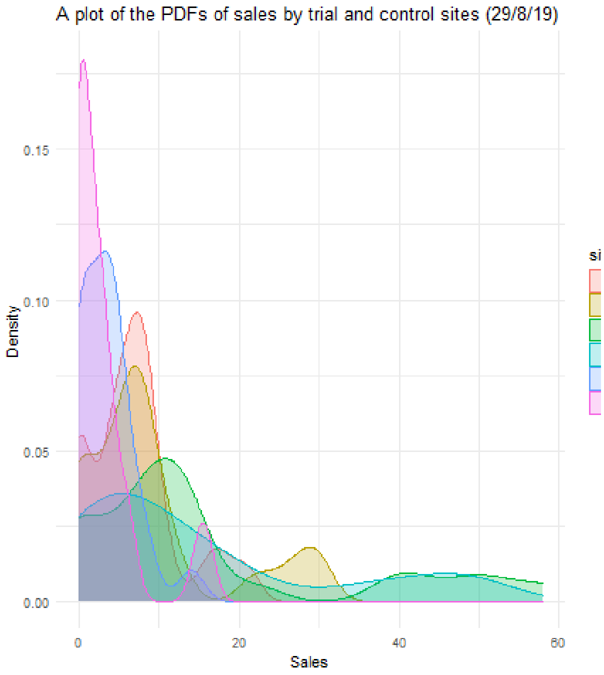


Figure 3: A plot of the PDFs for the 6 sites (3 trial sites and 3 control sites) for 29/8/19

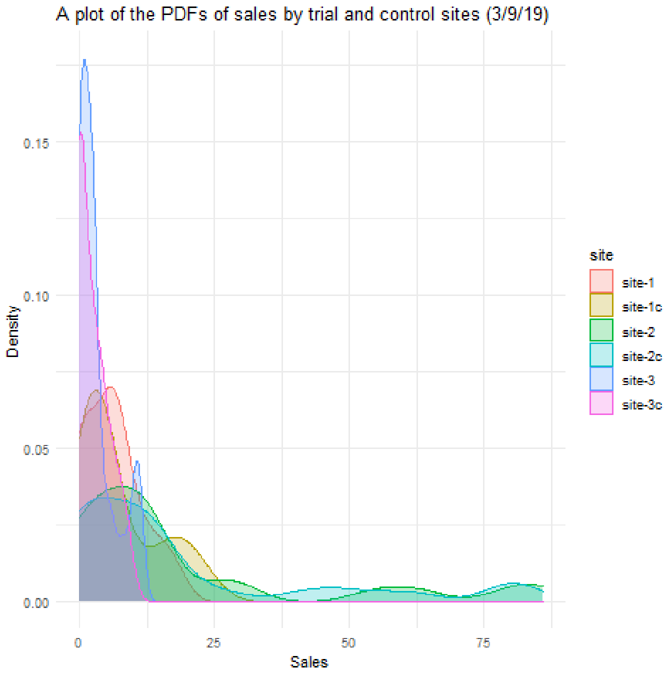


Figure 4: A plot of the PDFs for the 6 sites (3 trial sites and 3 control sites) for 3/9/19

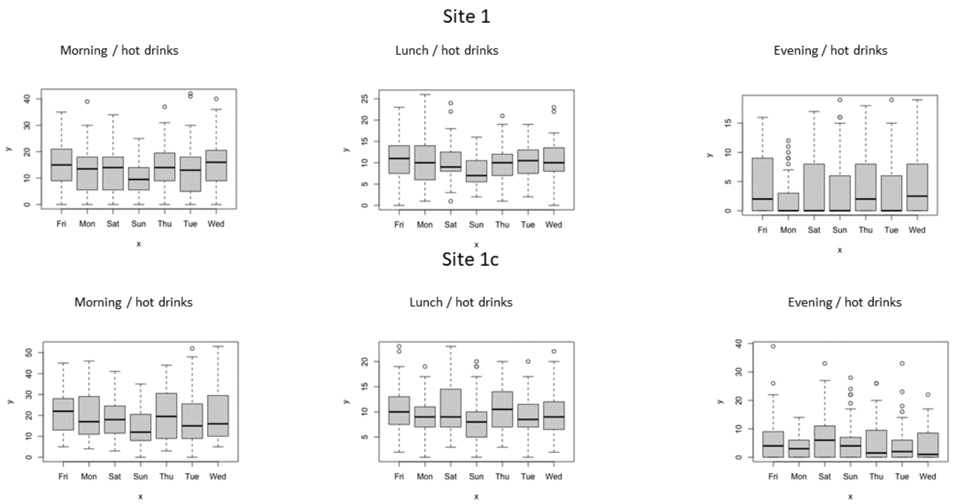


Figure 5: Box plots of the hot drinks sales for site 1 and its control by day and day part for a twelve weeks period

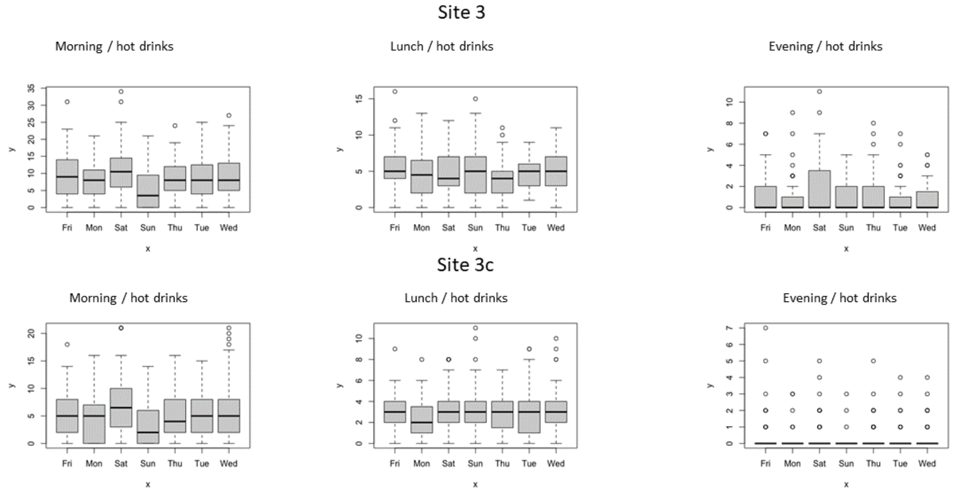


Figure 6: Box plots of the hot drinks sales for site 3 and its control by day and day part for a twelve weeks period

# 2 Model comparisons for product categories

| | ZI-NB GLMM | ZI-NB GLM | ZI-P GLMM | ZI-P GLM |
|---|---|---|---|---|
| **Fixed Effects** | | | | |
| Intercept | 2.46866 (0.38204)*** | 2.70733 (0.11466)*** | 2.49292 (0.37677)*** | 2.79648 (0.10899)*** |
| factor(month)November | 0.07674 (0.03607)* | 0.05692 (0.06059) | 0.07220 (0.03588)* | 0.03163 (0.05719) |
| factor(dow)Mon | -0.11969 (0.06427). | -0.00111 (0.10714) | -0.12473 (0.06354)* | -0.05708 (0.09915) |
| factor(dow)Sat | 0.09184 (0.05729) | 0.15452 (0.09876) | 0.08301 (0.05678) | 0.10980 (0.09249) |
| factor(dow)Sun | -0.15160 (0.06720)* | -0.06083 (0.11279) | -0.16070 (0.06684)* | -0.10243 (0.10429) |
| factor(dow)Thu | 0.06582 (0.05948) | 0.02358 (0.10196) | 0.04916 (0.05916) | -0.02342 (0.09548) |
| factor(dow)Tue | -0.07262 (0.06120) | -0.01052 (0.10552) | -0.08075 (0.06170) | -0.05187 (0.09686) |
| factor(dow)Wed | 0.01340 (0.05949) | 0.04073 (0.10305) | 0.00264 (0.05890) | -0.01446 (0.09537) |
| temperature | -0.01717 (0.01242) | 0.00947 (0.02047) | -0.01823 (0.01238) | 0.00652 (0.01943) |
| factor(daypart)Lunch | 0.31885 (0.29454) | 0.28911 (0.07539)*** | 0.32334 (0.28618) | 0.29378 (0.07055)*** |
| factor(daypart)Morning | 0.66949 (0.24367)** | 0.51168 (0.06147)*** | 0.66779 (0.23721)** | 0.44960 (0.05807)*** |
| factor(temp_band)11-15 | -0.10104 (0.12159) | -0.32488 (0.19681). | -0.09113 (0.12074) | -0.25903 (0.18705) |
| factor(temp_band)6-10 | 0.01972 (0.07102) | -0.11112 (0.11799) | 0.02134 (0.07056) | -0.09040 (0.11278) |
| **Zero-Inflated Effects** | | | | |
| Intercept | -2.5390 (0.3778)*** | -2.7675 (0.4840)*** | -2.5018 (0.3669)*** | -2.5146 (0.3799)*** |
| factor(dow)Mon | -1.9173 (2.0565) | -0.07416 (0.64577) | -1.3718 (1.1709) | -0.2715 (0.5557) |
| factor(dow)Sat | -1.6497 (0.9568). | -0.59682 (0.72402) | -1.6248 (0.9217). | -0.8117 (0.6437) |
| factor(dow)Sun | -0.8109 (0.7143) | -0.47344 (0.75985) | -0.8007 (0.6918) | -0.5883 (0.6430) |
| factor(dow)Thu | -3.2196 (3.3690) | -2.1457 (1.6024) | -2.8148 (2.2078) | -1.9497 (1.1174). |
| factor(dow)Tue | -16.4083 (2329.6678) | -1.7210 (1.3020) | -3.6826 (4.9808) | -1.5322 (0.8912). |
| factor(dow)Wed | -19.1612 (4869.0558) | -2.8416 (2.5245) | -17.6148 (2210.9938) | -2.4964 (1.3812). |
| **Random Effects** | | | | |
| hour (SD/Variance) | 0.2146 (0.4633) | NA | 0.2024 (0.4499) | NA |
| site (SD/Variance) | 0.3324 (0.5765) | NA | 0.3255 (0.5705) | NA |
| **Model Fit** | | | | |
| AIC | 5530.1 | 6330.9 | 5528.7 | 6299.0 |
| BIC | 5637.8 | 6429.2 | 5636.3 | 6397.3 |

Table 1: Model comparison (hot drinks)

| | ZI-NB GLMM | ZI-NB GLM | ZI-P GLMM | ZI-P GLM |
|---|---|---|---|---|
| **Fixed Effects** | | | | |
| Intercept | 3.04899 (0.26131) *** | 3.10331 (0.09113) *** | 3.051319 (0.249220) *** | 3.013634 (0.087944) *** |
| factor(month)November | -0.01849 (0.03634) | -0.07202 (0.05309) | -0.004651 (0.033458) | -0.033974 (0.046395) |
| factor(dow)Mon | -0.36091 (0.06240) *** | -0.40633 (0.09036) *** | -0.371560 (0.059036) *** | -0.296176 (0.081663) *** |
| factor(dow)Sat | -0.04858 (0.05981) | -0.10936 (0.08670) | -0.147318 (0.055056) ** | -0.052893 (0.076326) |
| factor(dow)Sun | -0.21628 (0.06412) *** | -0.25848 (0.09337) ** | -0.238991 (0.059387) *** | -0.189244 (0.082555) * |
| factor(dow)Thu | 0.04373 (0.05904) | 0.10667 (0.08627) | -0.017271 (0.053604) | 0.012033 (0.076917) |
| factor(dow)Tue | -0.24142 (0.06006) *** | -0.28548 (0.08791) ** | -0.242924 (0.054822) *** | -0.174690 (0.076127) * |
| factor(dow)Wed | -0.12839 (0.06018) * | -0.18194 (0.08741) * | -0.136545 (0.055404) * | -0.116196 (0.078086) |
| temperature | 0.02061 (0.01269) | 0.02194 (0.01770) | 0.021237 (0.011978) . | 0.037252 (0.016624) * |
| factor(daypart)Lunch | 0.09560 (0.21251) | 0.07772 (0.06343) | 0.112074 (0.210249) | 0.131914 (0.052934) * |
| factor(daypart)Morning | -0.35963 (0.17562) * | -0.27052 (0.05125) *** | -0.339052 (0.174512) . | -0.328306 (0.050175) *** |
| factor(temp_band)11-15 | -0.01944 (0.12066) | 0.17636 (0.16970) | -0.012925 (0.113036) | -0.007654 (0.158057) |
| factor(temp_band)6-10 | -0.05049 (0.07438) | 0.01285 (0.10526) | -0.036913 (0.069543) | -0.068120 (0.098122) |
| **Zero-Inflated Effects** | | | | |
| Intercept | -2.2794 (0.3205) *** | -2.2844 (0.3223) *** | -2.2919 (0.3235) *** | -2.2778 (0.3203) *** |
| factor(dow)Mon | -0.8219 (0.5588) | -0.9997 (0.6350) | -0.8106 (0.5609) | -1.1524 (0.7140) |
| factor(dow)Sat | -2.8666 (1.5734) . | -2.8538 (1.5635) . | -18.0941 (3635.4547) | -2.7737 (1.4444) . |
| factor(dow)Sun | -0.6214 (0.5656) | -0.5719 (0.5581) | -0.7651 (0.6127) | -0.5987 (0.5648) |
| factor(dow)Thu | -2.8161 (1.5123) . | -2.7584 (1.4392) . | -17.2412 (2683.6850) | -3.0694 (1.9591) |
| factor(dow)Tue | -17.0969 (1982.9246) | -2.1972 (1.0907) * | -20.1822 (7641.7098) | -2.2362 (1.1267) * |
| factor(dow)Wed | -2.5409 (1.1381) * | -3.0340 (1.8313) . | -2.7810 (1.4441) . | -3.1744 (2.1165) |
| **Random Effects** | | | | |
| hour (Variance) | 0.1103 | | 0.1086 | |
| site (Variance) | 0.1451 | | 0.1278 | |
| **Model Fit** | | | | |
| AIC | 5612.5 | 6138.4 | 5613.1 | 6121.5 |
| BIC | 5720.2 | 6236.8 | 5720.8 | 6219.8 |

Table 2: Model comparison (cold)

| | ZI-NB GLMM | ZI-NB GLM | ZI-P GLMM | ZI-P GLM |
|---|---|---|---|---|
| **Fixed Effects** | | | | |
| Intercept | 3.74314 (0.32910) *** | 3.60201 (0.11185) *** | 3.69902 (0.31266) *** | 3.74672 (0.09531) *** |
| factor(month)November | -0.04902 (0.03869) | -0.14141 (0.06623) * | -0.004976 (0.03260) | -0.11693 (0.04973) * |
| factor(dow)Mon | -0.35524 (0.06617) *** | -0.31449 (0.11149) ** | -0.29988 (0.05616) *** | -0.25416 (0.08768) ** |
| factor(dow)Sat | -0.11488 (0.06320) . | -0.14145 (0.10568) | -0.18776 (0.05293) *** | -0.06080 (0.08372) |
| factor(dow)Sun | -0.44681 (0.06801) *** | -0.42916 (0.11425) *** | -0.37677 (0.05957) *** | -0.44380 (0.09643) *** |
| factor(dow)Thu | 0.10403 (0.06302) . | 0.09626 (0.10547) | 0.01334 (0.05150) | 0.10441 (0.08213) |
| factor(dow)Tue | -0.27852 (0.06395) ** | -0.33591 (0.10774) ** | -0.23078 (0.05262) *** | -0.19963 (0.08096) * |
| factor(dow)Wed | -0.11969 (0.06402) . | -0.14492 (0.10760) | -0.11977 (0.05303) * | -0.07943 (0.08429) |
| tempreture | 0.01628 (0.01338) | 0.06240 (0.02145) ** | 0.00881 (0.01160) | 0.05024 (0.01796) ** |
| factor(daypart)Lunch | 0.10359 (0.33912) | 0.10268 (0.07779) | 0.20607 (0.33221) | 0.09859 (0.05658) |
| factor(daypart)Morning | -0.60033 (0.28014) * | -0.30285 (0.06209) *** | -0.49835 (0.27528) . | -0.58244 (0.05601) *** |
| factor(temp_band)11-15 | -0.09557 (0.12691) | -0.10619 (0.20413) | -0.05564 (0.10842) | -0.17739 (0.17382) |
| factor(temp_band)6-10 | -0.01450 (0.07815) | -0.07925 (0.12468) | 0.00389 (0.06580) | -0.11568 (0.10679) |
| **Zero-Inflated Effects** | | | | |
| Intercept | -2.3727 (0.3314) *** | -2.4005 (0.3400) *** | -2.3836 (0.3347) *** | -2.3782 (0.3331) *** |
| factor(dow)Mon | -0.4293 (0.5255) | -0.4714 (0.5559) | -0.6457 (0.5983) | -0.5766 (0.5845) |
| factor(dow)Sat | -15.0868 (881.7603) | -2.9419 (1.9097) | -20.9668 (11584.7147) | -2.6566 (1.4260) . |
| factor(dow)Sun | -1.3438 (0.7990) . | -1.0242 (0.7217) | -2.0248 (1.2981) | -1.6052 (1.2088) |
| factor(dow)Thu | -4.5065 (11.2532) | -1.8997 (1.0144) . | -20.8437 (11609.7811) | -1.7939 (0.9069) * |
| factor(dow)Tue | -16.0810 (916.6792) | -16.9993 (1471.1355) | -21.0021 (10794.5642) | -16.9573 (1424.6262) |
| factor(dow)Wed | -1.7039 (0.8143) * | -1.9173 (1.0056) . | -15.0818 (1512.7022) | -1.8713 (0.9497) * |
| **Random Effects** | | | | |
| hour (Variance) | 0.2875 | | 0.2771 | |
| site (Variance) | 0.1877 | | 0.1632 | |
| **Model Fit** | | | | |
| AIC | 6530.7 | 7302.5 | 6427.9 | 7208.0 |
| BIC | 6638.4 | 7400.9 | 6535.6 | 7306.3 |

Table 3: Model comparison (sandwiches)
